# Stent-Assisted Coiling Using Leo+ Baby Stent: Immediate and Mid-Term Results

**DOI:** 10.1007/s00062-020-00904-3

**Published:** 2020-05-08

**Authors:** Hannes Luecking, Tobias Struffert, Philipp Goelitz, Tobias Engelhorn, Sebastian Brandner, Joji B. Kuramatsu, Stefan Lang, Manuel Schmidt, Arnd Doerfler

**Affiliations:** 1 Department of Neuroradiology, University of Erlangen-Nuremberg, Schwabachanlage 6, 91054 Erlangen, Germany; 2 Department of Neurosurgery, University of Erlangen-Nuremberg, Erlangen, Germany; 3 Department of Neurology, University of Erlangen-Nuremberg, Erlangen, Germany; 4 Department of Neuroradiology, University of Giessen, Giessen, Germany

**Keywords:** Low profile stent, Braided stent, Cerebral aneurysm, Coiling, Bifurcation aneurysm

## Abstract

**Background:**

Stent-assisted coiling is well-established for treatment of cerebral aneurysms. The technique enables treatment of wide-neck, bifurcation and recurrent aneurysms with high packing rates. While described in extenso for laser cut stents, the results of patients treated with the Leo+ Baby (Balt, Montmorency, France) braided microstent are presented.

**Material and Methods:**

Patients were included if treated with a Leo+ Baby and with digital subtraction angiography (DSA) follow-up available of at least 6 months. Data were evaluated for successful deployment, aneurysm occlusion according to the modified Raymond-Roy classification (MRRC), stent patency and procedure-related morbidity and mortality.

**Results:**

A total of 81 patients were included and Leo+ Baby deployment was successful in all cases. Coils were used in 80 cases. In 1 case 2 stents were used stent-in-stent without additional coiling. Initial aneurysm occlusion rates were MRRC_i_1 51.9%, MRRC_i_2 11.1%, MRRC_i_3a 24.7% and MRRC_i_3b 12.3%. Occlusion rates after 6 months were MRRC_6m_1 78.9%, MRRC_6m_2 3.9%, MRRC_6m_3a 6.6% and MRRC_6m_3b 10.5%. Procedure-related morbidity was 1 case of acute stent thrombosis successfully treated with tirofiban and 1 case with transient hemiparesis due to stent thrombosis after 4 months. There was 1 case of coil-associated subarachnoid hemorrhage (SAH) which caused prolonged hospitalization. No procedure-related mortality was observed.

**Conclusion:**

The results confirm that stent-assisted coiling with the Leo+ Baby stent is safe and efficient for treatment of wide neck or recurrent cerebral aneurysms. Spontaneous progressive aneurysm occlusion over 6 months supports the theory of considerable flow-modulating effects of Leo+ Baby.

## Introduction

In the last decade the possibilities for endovascular treatment of cerebral aneurysms (CA) have been considerably extended and studies have proved lower morbidity and mortality for coiling than for surgical clipping [1, 2]; however, many aneurysms are still challenging to treat, especially if very small, wide neck, located at a bifurcation or at distant parent vessels [3].

**Fig. 1:**
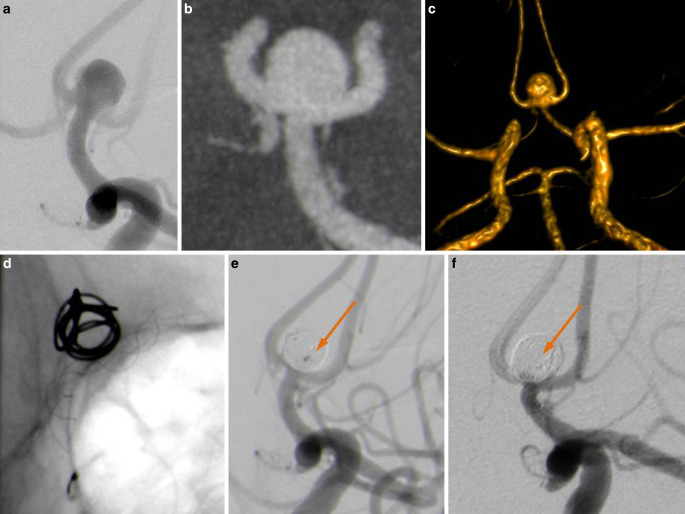
Wide-neck Acom aneurysm involving both A2 branches (**a**, **b**). FD-CT with iv contrast (**c**) shows dominant feeding of both A2 branches from the left side. Note the two helical marker strands (**d**) indicating complete opening of the stent. *Arrow* in **e** marks residual central inflow (MRRC3a) by the end of the procedure which had disappeared at follow-up (**f**). **a**, **b** and **d–f** show DSA images

If plain or balloon-assisted coiling is not feasible, the additional use of new generation braided microstents (BMS) may represent an option for those aneurysms [4]. The braided (thus kind of closed cell) design helps to prevent coil dislocation and is assumed to provide a blood flow-modulating effect like a flow-diverting stent (FDS) device [5, 6]. Free to move strut intersections are responsible for good compliance of these stents, which can facilitate adaptation to complex vessel anatomies. Currently used devices, such as the Leo+ Baby (Balt, Montmorency, France) or the Lvis jr. (MicroVention, Tustin, CA, USA) are equipped with two radiopaque strands arranged as a double helix to assess stent opening under fluoroscopy (Fig. 1d).

Beside the jailing technique the higher porosity of BMS compared to FDS enables a microcatheter to be advanced through the mesh after deployment. The low stent profile enables usage of 0.017 in. microcatheters (MC) for stent delivery, which facilitates access to small and distal vessels. Depending on the stent diameter BMS deployment is possible in parent vessel sizes down to 1.5 mm in diameter. As with laser-cut stents complex constructs like T‑stenting or kissing stents are attainable [7, 8] as well. Due to the braided design it is possible to re-capture a BMS if already deployed up to 80% of the total stent lenght. This enables precise positioning or if necessary, complete removal and exchange of the device.

Data on the use of these devices are still rarely documented in literature. This study reports on a single center experience and mid-term results in a series of 81 aneurysms treated by a BMS (Leo+ Baby) stent-assisted coiling.

## Material and Methods

We reviewed our data base retrospectively and included all patients treated by a Leo+ Baby stent since April 2014. Patients were included if a follow-up angiogram within at least 6 months was available.

### Medication and Preparation

Based on our institutional standards patients were prepared with 75 mg of clopidogrel and 100 mg of aspirin (ASA) 7 days prior to treatment procedure. Upon deployment 5000 IU of heparin was administered. All procedures were performed with the patient under general anesthesia. There was 1 case with acute subarachnoid hemorrhage (SAH) who was administered 250 mg of ASA and 5000 IU of heparin prior to stent deployment and 300 mg of clopidogrel via a gastric tube with a 2 h interval.

Depending on follow-up clopidogrel was stopped routinely after 6 weeks whereas aspirin was continued until at least 1.5 years posttreatment.

In our institution platelet function testing (Multiplate® Analyzer Array (Roche Deutschland Holding GmbH; Grenzach-Wyhlen, Germany) is available from the central laboratory) is not performed routinely for patients scheduled for stenting, as opposed to patients planned for flow-diverters.

### Treatment Procedure

Prior to treatment all patients underwent diagnostic catheter angiography for treatment planning including 3‑D rotational angiography. All treatments were performed on a biplane flat detector angiographic system (Artis zee Biplane System, Siemens AG, Healthineers, Forchheim, Germany). After the treatment procedure flat-detector computed tomography (FD-CT) is routinely acquired to rule out procedure-related hemorrhage.

Leo+ Baby stent is delivered via a 0.017″ microcatheter (MC). Usage of a Vasco + 10 MC (Balt) is recommended by the manufacturer, although use of any MC of the same inner diameter is possible. Vasco + 10 is relatively stiff to facilitate re-capturing the stent, which—on the other hand—sometimes causes difficulties to probe tortuous vessels or sharp angels. In those cases an Echelon 10 MC (Medtronic, Irvine, CA, USA) was used.

Bioactive Cerecyte™ coils (Codman Neuro; Raynham, MA, USA) were used according to the aneurysm size. Coil packing as dense as possible was always attempted. If applicable, the MC was jailed within the aneurysm lumen prior to deployment (*n* = 72) or after deployment through the stent struts (*n* = 8).

### Imaging Follow-up

As a standard procedure magnetic resonance imaging (MRI), diffusion and perfusion weighted imaging (DWI/PWI), time-of-flight and contrast-enhanced angiography, T2w FLAIR (fluid attenuated inversion recovery), T2* and contrast enhanced T1w sequences and contrast-enhanced FD-CT (source images, multiplane and volume rendering technique (VRT) reconstructions) [9, 10] were acquired 2 weeks and 3 months after intervention to monitor brain parenchyma (e.g. silent ischemia), BMS configuration and BMS lumen. As the current gold standard DSA was performed at 6 months after treatment.

### Imaging Evaluation

Follow-up imaging was evaluated by two experienced interventionists in consensus reading.

The modified Raymond-Roy classification (MRRC) [11] was used for occlusion assessment in DSA, FD-CT and MRA (MRRC1 = complete occlusion; MRRC2 = neck remnant (dog ear); MRRC3a = residual aneurysm, without contact to aneurysm wall; MRRC3b = residual aneurysm, with contact to aneurysm wall).

Wall adaptation, BMS configuration, endothelial hyperplasia and in-stent-thrombosis were evaluated in DSA and FD-CT. Cut-off for report of endothelial hyperplasia was 20% of the stent lumen. MRI was evaluated for indirect signs of BMS malfunction (e.g. by time-to-peak delay in PWI).

MRI was also analyzed for asymptomatic pathologic changes of brain tissue (e.g. post-ischemic lesions, inflammation, etc.) and hemorrhage.

### Clinical Follow-up

Patients were examined by a neurologist or neurosurgeon at admission, after the procedure, and at discharge. Assessment criteria were procedure-related neurologic deficits or death. Furthermore, all patients were clinically assessed at the follow-up visits.

## Results

All data were analyzed and interpreted by two experienced (11 and 20 years) interventionists.

### Patient Inclusion

A total of 81 consecutive patients treated with a Leo+ Baby stent were identified.

Of the patients 5 refused to have DSA follow-up and underwent follow-up with FD-CT and MRI. In these 5 cases the aneurysm was rated as MRRC1 (complete occlusion) after 6 months.

Mean dome-to-neck ratio was 1.06 ± 0.22, indicating that only wide neck aneurysms had been selected to justify stent-assisted coiling.

### Technical Procedure

Deployment of the BMS (*n* = 86) was successful in all cases, although in 3 cases the stent chosen was too short and a second stent had to be attached at the proximal end. In 2 cases 2 BMSs were used for T‑stenting. In 1 case of a fusiform aneurysm 2 BMS were used stent-in-stent to achieve a flow-diverting effect (MRRC_i_ = 3b; MRRC_6M_ = 1). In 8 cases the aneurysm was coiled through the stent mesh whereas in 72 cases the MC was jailed.

In 3 cases the stent-carrying MC needed to be looped through the aneurysm to probe the distal orifice of the parent vessel and a stent-retriever was used to straighten the MC. These interventions were otherwise unremarkable. In 1 case the jailed MC popped out of the aneurysm during stent deployment (aneurysm mean size 2.7 mm) so that coiling was done in a second intervention 6 weeks later after the stent was assumed to be integrated into the vessel wall.

Table 1 provides an overview of demographic data and aneurysm anatomy.

**Table 1:** Demographic data and aneurysm anatomy of all patients initially included

*Demography*	Overall	Female	Male
Sex	81	55	26
Age (years)	58.1 ± 11.9 (16–85)	57.9 ± 10.2 (16–85)	58.7 ± 12.7 (35–77)
*Aneurysm anatomy*			
Aneurysm size (mm)	4.6 ± 2.4 (2.0–12.5)		
Dome/neck ratio	1.06 ± 0.22		
Location	ACA: 43; MCA: 18; PCA/BA: 14; PCA: 5; PICA: 1		

*ACA/MCA/PCA* anterior/middle/posterior cerebral artery, *BA* basilar artery, *PICA* posterior inferior cerebellar artery

### Procedural Safety

We observed 1 patient with acute in-stent thrombosis during the procedure. The thrombosis was successfully treated by tirofiban infusion (a simplified scheme for a package size of 250 ml/50 µg/ml is used with an initial dose of 25 ml at a flow rate of 50 ml/h, with the remaining 225 ml being administered at a flow rate of 9 ml/h for approximately 23 hours). Postprocedural MRI with DWI showed no diffusion restriction in this case. There were no further acute complications requiring particular treatment. By the end of the procedure all stents were patent. Good wall adaptation at the proximal and distal landing zones was documented in all cases at least in 2D DSA or, if not impaired by hardening artifacts, also in 3D imaging. Postprocedural MRI (*n* = 78, 2 cases with cardiac pacemaker, 1 not done due to clinical conditions after SAH) was unremarkable in 64 cases, 11 patients had 1–3 punctual DWI lesions, 3 patients had >3 DWI lesions. No territorial infarct was recorded. All patients were non-symptomatic for the DWI lesions.

There was 1 case of new hemorrhage in postprocedural non-enhanced FD-CT with fluoroscopy documented coil perforation, which was judged as not directly associated with the Leo+ Baby. The patient completely recovered. Yet, this led to prolonged hospitalization.

There was no procedure-associated mortality.

### Initial Occlusion Rate

In the 81 patients initially treated MRRC_i_1 was assessed in 35 cases (51.9%) by the end of the procedure. MRRC_i_2 was found in 5 cases (11.1%), MRRC_i_3a in 16 cases (24.7%) and MRRC_i_3b in 6 cases (12.3%).

Table 2 summarizes the procedure and follow-up results.

**Table 2:** Occlusion rates and adverse events during procedure and after 6 months of follow-up

*Procedure (n* *=* *81)*	
Stents used	86
Stent alone	1 (stent-in-stent)
Stent-assisted coiling	80 (*n* = 1 T-stenting, *n* = 3 > 1 stent used)
Jailing/through mesh	74/6
Initial occlusion rate	MRRC_i_1: *n* = 42; 51.9%/MRRC_i_2: *n* = 9; 11.1%/MRRC_i_3a: *n* = 20; 24.7%/MRRC_i_3b: *n* = 10; 12.3%
Adverse events	Acute stent thrombosis: *n* = 1 (1.2%)
	DWI/FLAIR lesions (non-symptomatic): *n* = 14 (17.3%)
	DWI/FLAIR lesions (symptomatic): none
	Hemorrhage: *n* = 1 (1.2%, coil-associated)
Mortality	None
*Follow-up (n* *=* *76)*	
Interval	7.2 ± 1.6 (5–11) months
Modality	DSA: *n* = 76; FD-CT + MRI: *n* =5
Occlusion DSA *n* = 76	MRRC_6M_1: *n* = 60; 78.9%/MRRC_6M_2: *n* = 3; 3.9%/MRRC_6M_3a: *n* = 5; 6.6%/MRRC_6M_3b: *n* = 8; 10.5%
Adverse events	Delayed stent thrombosis: *n* = 1 (1.8%)
	DWI/FLAIR lesions (non-symptomatic): none
	DWI/FLAIR lesions (symptomatic): 1 (1.8%)
	Hemorrhage: none
	Endothelial hyperplasia: *n* = 2 (3.5%)
	Recurrence: none
Mortality	None

### Follow-up

DSA follow-up was available in 76 patients. Mean time interval to the procedure was 7.2 ± 1.6 months (range 5–11 months). After 6 months complete aneurysm occlusion MRRC_6M_1 was assessed in 60 cases (78.9%) whereas MRRC_6M_2 was found in 2 cases (3.9%), MRRC_6M_3a in 5 cases (6.6%) and MRRC_6M_3b in 8 cases (10.5%). The 5 missing patients refused to have DSA for follow-up; however, they underwent non-invasive follow-up with MRI and contrast-enhanced FD-CT which showed complete occlusion in all 5 patients.

DSA showed no stent migration or other changes in device configuration. All stents were patent except for 1 case. A 16-year-old girl was admitted to a hospital nearby 4 months after treatment of a proximal posterior cerebral artery (PCA) aneurysm for severe headaches and a mild sensorimotor hemiparesis. MRI there showed a complete occlusion of the stent and aneurysm. The P1 segment was occluded and an infarct 5 mm in size of the left (ipsilateral) thalamus was present. Stent thrombosis was confirmed by DSA. The symptoms dissolved until discharge. When the patient was admitted to our department for routine follow-up after 6 months she had almost completely recovered.

Endothelial hyperplasia was found in 2 cases within the first 3 months without hemodynamic effect in PWI. Yet, dual platelet inhibition had been extended until the angiography for those cases.

MRI after 6 months was available in all patients (*n* = 79) except in two cases (cardiac pacemaker). Except for the 16-year-old patient already mentioned there were no new DWI or FLAIR lesions nor was there any further case of hemorrhage.

Spontaneous subarachnoid hemorrhage or mortality was not observed during follow-up.

**Fig. 2:**
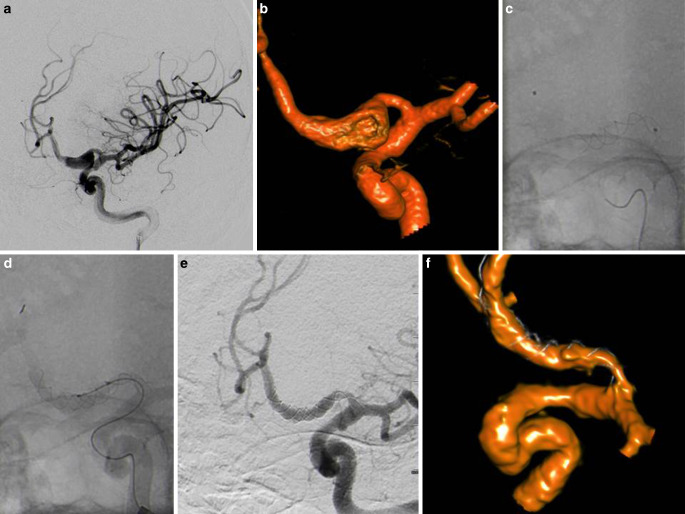
Incidental dissecting aneurysm of the left A1-segment in DSA and FD-CT with intraarterial contrast application (**a**, **b**). Placement of two overlapping Leo+ Baby (Balt, Montmorency; 2.5 × 25 and 2.5 × 18 mm; **c**, **d**). Follow-up after 5.6 months shows complete occlusion in DSA and FD-CT with intraarterial contrast application (**f**). FD-CT is of limited quality due to movement artifacts

Table 1 provides an overview over demographic data and aneurysm anatomy. Table 2 summarizes the procedure and follow-up results. For a graphic presentation of initial and 6 months occlusion rates see Fig. 2.

## Discussion

Treatment of wide-neck or recurrent cerebral aneurysms can still be challenging if located at the periphery or if a bifurcation is involved. Balloon-assisted coiling is an option in certain situations but bears a higher risk of retreatment [12, 13]. In addition to laser-cut stents, flow-diverters and bifurcation devices (stent-like or intrasaccular) a number of low-profile braided stents are available designed to treat peripheral or otherwise difficult to treat aneurysms. We report on our experience with the Leo+ Baby braided stent in a series of 81 patients.

### Procedure

Leo+ Baby deployment and aneurysm coverage was finally successful in all cases, although in 3 cases a second stent was necessary to completely cover the aneurysm.

Due to their particular construction with free to move strut intersections Leo+ stents are able to open considerably (up to 0.6 mm) wider than their nominal diameter while their length decreases substantially if the chosen stent is too small in relation to the parent vessel diameter. This property may help to more reliably achieve complete wall adaptation as unintended minor undersizing can be compensated. Also, this property may allow a better reconstruction of the aneurysm neck as deliberate bulging of the stent, e.g. at wide bifurcations, can be achieved.

For planning we recommend consulting all modalities available, i.e. 2D and 3D data from DSA to determine a BMS size equal to or slightly smaller than the maximum parent vessel diameter. If the length measurement, which can most accurately be done be micro-wire, is between two sizes, it is our experience to rather choose a longer than a shorter size.

There was only one case of acute stent thrombosis which could be treated successfully by tirofiban intra-arterial injection without clinical sequelae. In our center patients to be treated with a BMS are not tested for drug response. It is our experience that thromboembolic complications are rare with these devices and there is even hint for no more complications in drug non-responders [14].

No territorial infarcts were observed. A total of 14/78 patients (17.9%) showed punctual DWI lesions in postinterventional MRI.

There was 1 case of procedure-related hemorrhage. This was verifiably caused by coil perforation and thus was assessed to be not associated with the stent itself. Yet, procedure-related hemorrhage in general can take a more unfavorable course in patients prepared with dual anti-platelet therapy. Furthermore, the risk of coil perforation in smaller aneurysms can be increased when using the jailing technique as a higher force can be applied with the MC retained between the stent and the vessel wall.

### Aneurysm Occlusion

Occlusion rates by the end of intervention were 60.5% MRRC_i_1 and 2, 26.3% MRRC_i_3a and 13.2% MRRC_i_3b. We noticed a shift in sufficient occlusion after 6 months to 82.8% (MRRC_6M_1: *n* = 60 (78.9%); MRRC_6M_2: *n* = 2 (3.9%)). The rate of MRRC_6M_3a and 3b decreased to 6.6 and 10.5%, respectively. In our study population none of the MRRC3a/b patients was scheduled for retreatment so far as all patients were at least still on aspirin and potential further spontaneous occlusion should be awaited.

**Fig. 3:**
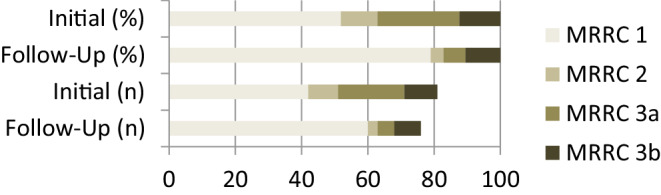
Shift of occlusion rates (absolute numbers) from treatment to 6 months follow-up

The shift from relatively low initial occlusion rates to high rates of >80% within several months otherwise is a typical feature and designated working principle of low-porosity flow diverter stents [15–18]. Although in our study group all patients were still at least on aspirin, we observed spontaneous complete occlusion from MRRC_i_2/3a/3b to MRRC_6M_1 in as many as 23.6% (*n* = 18) of patients. This included 1 case in which 2 overlapping Leo+ Baby were used without coiling for treatment of a fusiform 11 × 7 mm aneurysm (Fig. 3). An explanation is the higher metal coverage (smaller cell size) of the Leo+ Baby compared to other laser-cut and braided MSs—although it is still not comparable to typical FDSs [3, 19]. A smaller cell size works as a more effective scaffold for endothelial growth leading to a progressive sealing of the aneurysm neck [20, 21]. This can result in short-term to mid-term aneurysm occlusion. Also, it is possible to condense the stent struts at the aneurysm neck with braided devices, which helps to further increase metal coverage. A flow-diverting effect of BMS had already been discussed by Machi et al. [22] and Pumar et al. [23]. Neointimal thickness, on the other hand, is reported to be positively correlated with metal coverage [21]. This should be kept in mind in treatment of very small parent vessels.

A positive side effect of the lower porosity is an assumed lower risk of losing coils in treatment of very small aneurysms.

Djurdjevic et al. [24] recently reported on a series of 101 patients treated with Leo+ Baby. In their follow-up after 3–6 months they found complete aneurysm occlusion in 37.9%, residual neck in 25.3% and residual aneurysm in 36.8%. Although progressive occlusion occurred in 21.8% these occlusion rates are remarkably lower compared to our results. A relatively low rate of initial MRRC1 occlusion in their group of 24.7% and a rate of 15% and 17.2% of MRRC3a and MRRC3b, respectively, led us to the assumption that a somewhat loose coil packing might have been the cause for these results. Machi et al. reported on a series of 29 patients with initial occlusion rates of 55%, 27% and 10% (MRRC1, 2 and 3a + 3b, respectively), with follow-up occlusion rates of as much as 96% MRRC1 and 4% MRRC2. Aydin et al. [25] observed complete aneurysm occlusion after 7 months in 85.7% in 80 patients after initial complete occlusion of 75%.

### Delayed Complications

During the follow-up there was delayed stent thrombosis in 1 case although 3 months MRI and FD-CT was non-remarkable. In MRI a circumscribed 5 mm infarct of the left thalamus was found. Anamnesis yielded a continuous taking of ASS and clopidogrel. As this patient had almost co-dominant P1 and posterior communicating segments we assume that minor hemodynamic changes caused by the BMS placed in the P1 segment may have caused the stent thrombosis. The patient recovered from the mild sensorimotor hemiparesis until the regular follow-up after 6 months. We recorded no further ischemic events. Djurdjevic et al. found delayed ischemic complication in 4 patients (4%) although dual platelet inhibition was administered for at least 6 months with aspirin monotherapy thereafter for at least another 6 months. Yet, in two cases the patient’s drug compliance was reported to be uncertain.

Neither stent-associated hemorrhage nor mortality was recorded during the procedure or in the follow-up period. In the meta-analysis by Cagnazzo et al. an overall mortality of 1.1% out of 750 aneurysms treated with different kinds of microstents was reported.

### Limitations

The major limitation of this survey is the retrospective single-center design and constricted time interval. A long-term follow-up will be complemented as soon as the data will be available with special respect to stable occlusion and possible progressive occlusion.

## Conclusion

Our results confirm that stent-assisted coiling of complex and recurrent cerebral aneurysms using Leo+ Baby braided microstent goes along with high procedural safety and high rates of aneurysm occlusion after 6 months. We observed a high percentage of progressive occlusion and no case of recurrence, suggesting a relevant flow-modulating effect of this particular stent.
